# Cesarean section was not associated with mortality or morbidities advantage in very low birth weight infants: a nationwide cohort study

**DOI:** 10.1038/s41598-021-99563-8

**Published:** 2021-10-12

**Authors:** Jin Kyu Kim, Yun Sil Chang, Jong Hee Hwang, Myung Hee Lee, Won Soon Park

**Affiliations:** 1 Department of Pediatrics, Jeonbuk National University School of Medicine, Jeonju, Korea; 2 Department of Pediatrics, Samsung Medical Center, Sungkyunkwan University School of Medicine, Seoul, Korea; 3 Department of Pediatrics, Ilsan Paik Hospital, InJe University College of Medicine, Goyang, Korea; 4 Research and Statistic Center, Social Information Research Institute, Seoul, Korea; 5 Research Institute of Clinical Medicine of Jeonbuk National University-Biomedical Research Institute of Jeonbuk National University Hospital, Jeonju, Korea

**Keywords:** Risk factors, Neonatology

## Abstract

This study investigated the role of cesarean section (CS) in mortality and morbidity of very-low-birth-weight infants (VLBWIs) weighing less than 1500 g. This nationwide prospective cohort study of the Korean Neonatal Network consisted of 9,286 VLBWIs at 23–34 gestational weeks (GW) of age between 2013 and 2017. The VLBWIs were stratified into 23–24, 25–26, 27–28 and 29–34 GW, and the mortality and morbidity were compared according to the mode of delivery. The total CS rate was 78%, and was directly proportional to gestational age. The CS rate was the lowest at 61% in case of infants born at 23–24 GW and the highest at 84% in VLBWIs delivered at 29–34 GW. Contrary to the significantly lower total mortality (12%) and morbidities including sepsis (21%) associated with CS than vaginal delivery (VD) (16% and 24%, respectively), the mortality in the 25–26 GW (26%) and sepsis in the 27–28 GW (25%) and 29–34 GW (12%) groups were significantly higher in CS than in VD (21%, 20% and 8%, respectively). In multivariate analyses, the adjusted odds ratios (ORs) for mortality (OR 1.06, 95% CI 0.89–1.25) and morbidity including sepsis (OR 1.12, 95% CI 0.98–1.27) were not significantly reduced with CS compared with VD. The adjusted ORs for respiratory distress syndrome (1.89, 95% CI 1.59–2.23) and symptomatic patent ductus arteriosus (1.21, 95% CI 1.08–1.37) were significantly increased with CS than VD. In summary, CS was not associated with any survival or morbidity advantage in VLBWIs. These findings indicate that routine CS in VLBWIs without obstetric indications is contraindicated.

## Introduction

Recent advances in perinatal and neonatal intensive care medicine have led to a remarkable improvement in survival and morbidity of very-low-birth-weight infants (VLBWIs) weighing less than 1500 g^1–3^. However, whether the mode of delivery affects the outcome of VLBWIs is still debated^4–8^. The presumed benefits of planned cesarean section (CS) in VLBWIs are counterbalanced by the potential fetal and maternal complications associated with the operation^9^.

Despite the current lack of robust scientific evidence supporting the benefit of CS in VLBWIs, there has been an exponential increase in CS rates involving VLBWIs in recent years^1–3^. The resurgence in CS rate has rendered it virtually impossible to conduct new randomized controlled trials (RCTs) to determine the optimal mode of delivery of VLBWIs^10, 11^. Therefore, analyzing very large data sets from a population-based, comprehensive prospective observational cohort of neonatal networks is the best alternative to RCTs to validate the risk and benefits of CS in VLBWIs.

The Korean Neonatal Network (KNN) is an ongoing nationwide, multicenter, prospective and web-based cohort registry system of VLBWIs^12, 13^. To determine whether CS was associated with survival or morbidity advantage in VLBWIs, the 9286 eligible VLBWIs delivered at 23–34 gestational weeks (GW) registered in KNN were stratified into 23–24, 25–26, 27–28 and 29–34 GW groups. The current study compared the total and gestational age (GA)-stratified mortality and morbidity according to the delivery mode.

## Results

### Cesarean section rate

**Figure 1:**
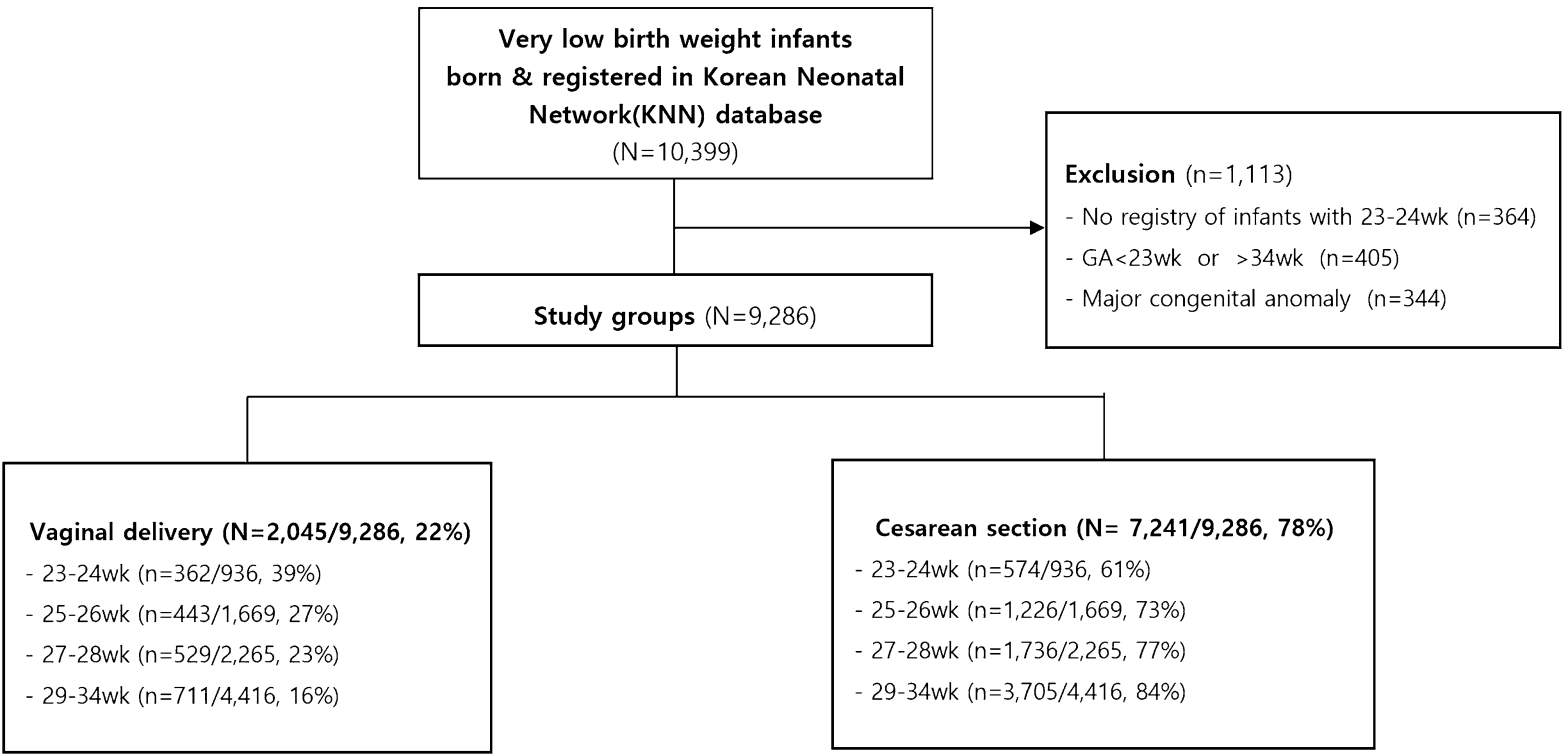
Flowchart outlining the study population. This study enrolled 10,399 VLBWIs. We excluded 1113 infants, and these infants were categorized into two groups based on the mode of delivery.

While the total CS rates were 78%, the CS rate was directly proportional to GA. The CS rates were 61%, 73%, 77% and 84% in case of VLBWIs delivered at 23–24 GW, 25–26 GW, 27–28 GW and 29–34 GW, respectively (Fig. 1, Table 1).

**Table 1:** Comparison of subjects’ demographic and perinatal characteristics.

Clinical characteristics	23–24 weeks (N = 936)		25–26 weeks (N = 1669)		27–28 weeks (N = 2265)		29–34 weeks (N = 4416)		Total (N = 9286)	
	VD	CS	VD	CS	VD	CS	VD	CS	VD	CS
	n = 362 (39%)	n = 574 (61%)	n = 443 (27%)	n = 1226 (73%)	n = 529 (23%)	n = 1736 (77%)	n = 711 (16%)	n = 3705 (84%)	n = 2045 (22%)	n = 7241 (78%)
Gestational age (weeks)	24^0/7^ ± 0^4/7^	241/7 ± 04/7*	26^0/7^ ± 0^4/7^	26^0/7^ ± 0^4/7^	28^0/7^ ± 0^4/7^	28^0/7^ ± 0^4/7^	30^4/7^ ± 1^3/7^	311/7 ± 14/7*	27^5/7^ ± 2^4/7^	290/7 ± 25/7*
Birth weight (g)	654.9 ± 108.3	649.0 ± 117.6	874.6 ± 134.3	818.8 ± 166.2*	1136.3 ± 161.0	1054.0 ± 209.3*	1335.1 ± 136.4	1246.8 ± 202.9*	1063.5 ± 288.9	1080.7 ± 279.3*
1-min Apgar score	2.9 ± 1.7	2.8 ± 1.7	4.0 ± 1.8	3.5 ± 1.7*	4.9 ± 1.8	4.3 ± 1.8*	6.0 ± 1.8	5.4 ± 1.8*	4.7 ± 2.1	4.6 ± 2.0
5-min Apgar score	5.0 ± 2.0	5.2 ± 2.0	6.2 ± 1.9	6.0 ± 1.8*	7.0 ± 1.6	6.7 ± 1.7*	7.7 ± 1.4	7.5 ± 1.5*	6.7 ± 1.9	6.9 ± 1.8*
Male sex, n (%)	181 (50)	290 (51)	240 (54)	650 (53)	293 (55)	922 (53)	361 (51)	1776 (48)	1075 (53)	3638 (50)
Small for gestational age, n (%)	37 (10)	99 (17)*	24 (5)	194 (16)*	14 (3)	223 (13)*	115 (16)	1561 (42)*	190 (9)	2077 (29)*
Antenatal steroid, n (%)	246 (68)	469 (84)*	337 (78)	1031 (86)*	420 (81)	1471 (86)*	553 (79)	2942 (81)	1556 (77)	5913 (83)*
Multiple pregnancy, n (%)	101 (28)	217 (38)*	86 (19)	419 (34)*	91 (17)	632 (36)*	202 (28)	1507 (41)*	480 (23)	2775 (38)*
Inborn, n (%)	345 (95)	557 (97)	419 (95)	1193 (97)*	489 (92)	1682 (97)*	677 (95)	3633 (98)*	1930 (94)	7065 (98)*
Maternal GDM, n (%)	9 (2)	18 (3)	34 (8)	76 (6)	53 (10)	140 (8)	72 (10)	350 (9)	168 (8)	584 (8)
Pregnancy induced hypertension, n (%)	9 (2)	40 (7)*	11 (2)	137 (11)*	9 (2)	292 (17)*	57 (8)	1217 (33)*	86 (4)	1686 (23)*

*VD* vaginal delivery, *CS* Cesarean section, *GDM* gestational diabetes mellitus.

*p < 0.05 compared with VD; Values are expressed as mean ± standard deviation or number (%).

### Demographic and perinatal characteristics

Demographic and perinatal characteristics of the VLBWIs according to the delivery mode in the total and GA-stratified subgroups are shown in Table 1. GA, male sex, antenatal steroid use, multiple pregnancies, inborn babies, maternal gestational diabetes mellitus (GDM) and pregnancy-induced hypertension (PIH) were significantly higher with CS, whereas chorioamnionitis was significantly lower with CS than VD both in the total and GA-stratified subgroups. Contrary to significantly higher total birth weight and Apgar score at 5 min with CS than VD, these variables were significantly lower in case of a few GA-stratified subgroups.

### Mortality and morbidity according to delivery mode

Table 2 presents the mortality and morbidity based on the delivery mode of infants in the total and GA stratified subgroups. A significantly lower total mortality, respiratory distress syndrome (RDS) and sepsis were associated with CS than VD; however, these variables were significantly higher with CS than VD in some GA-stratified subgroups. Significantly lower symptomatic patent ductus arteriosus (PDA), bronchopulmonary dysplasia (BPD), periventricular leukomalacia (PVL) and necrotizing enterocolitis (NEC) with CS than VD were observed only in the total study population, but not in the GA-stratified subgroups. Severe intraventricular hemorrhage (IVH) was significantly higher with CS than in VD only in the 29–34 GW subgroup but not in the total group.

**Table 2:** Comparison of mortality and morbidities in the study population.

Clinical outcomes	23–24 weeks (N = 936)		25–26 weeks (N = 1669)		27–28 weeks (N = 2265)		29–34 weeks (N = 4416)		Total (N = 9286)	
	VD	CS	VD	CS	VD	CS	VD	CS	VD	CS
	n = 362 (39%)	n = 574 (61%)	n = 443 (27%)	n = 1226 (73%)	n = 529 (23%)	n = 1736 (77%)	n = 711 (16%)	n = 3705 (84%)	n = 2045 (22%)	n = 7241 (78%)
Mortality, n(%)	190 (52)	274 (48)	91 (21)	314 (26)*	34 (6)	153 (9)	13 (2)	105 (3)	328 (16)	846 (12)*
Air leak syndrome, n (%)	60 (17)	94 (16)	36 (8)	132 (11)	21 (4)	72 (4)	5 (1)	77 (2)*	122 (6)	375 (5)
Respiratory distress syndrome, n (%)	353 (98)	563 (98)	427 (96)	1204 (98)*	479 (91)	1637 (94)*	409 (58)	2341 (63)*	1668 (82)	5745 (79)*
Bronchopulmonary dysplasia (≥moderate), n (%)	138 (78)	237 (75)	200 (56)	543 (58)	162 (32)	566 (36)	88 (13)	533 (15)	588 (34)	1879 (29)*
Symptomatic patent ductus arteriosus, n (%)	171 (52)	280 (52)	202 (47)	598 (51)	178 (34)	647 (38)	95 (14)	666 (18)*	646 (33)	2191 (31)
Intraventricular hemorrhage (≥ Gr. III), n (%)	99 (33)	187 (36)	82 (19)	195 (17)	47 (9)	132 (8)	13 (2)	80 (2)	241 (12)	594 (8)*
Periventricular leukomalacia, n (%)	38 (13)	74 (15)	61 (14)	135 (12)	48 (9)	142 (8)	32 (5)	191 (5)	179 (9)	542 (8)*
Necrotizing enterocolitis (≥ stage 2), n (%)	67 (19)	95 (17)	54 (12)	142 (12)	38 (7)	122 (7)	19 (3)	103 (3)	178 (9)	462 (6)*
Neonatal sepsis, n (%)	153 (43)	226 (40)	162 (37)	411 (34)	105 (20)	432 (25)*	60 (8)	436 (12)*	480 (24)	1505 (21)*
Early-onset sepsis within 7 days of life, n (%)	49 (14)	51 (9)*	35 (8)	72 (6)	25 (5)	81 (5)	13 (1)	81 (2)	122 (6)	285 (4)*
Retinopathy of premature (op.), n (%)	106 (56)	159 (49)	79 (22)	225 (24)	18 (4)	104 (6)*	3 (1)	41 (1)	206 (12)	529 (8)*

*VD* vaginal delivery, *CS* Cesarean section, *op* operation.

*p < 0.05 compared with VD.

### Adjusted ORs for mortality and morbidity

**Figure 2:**
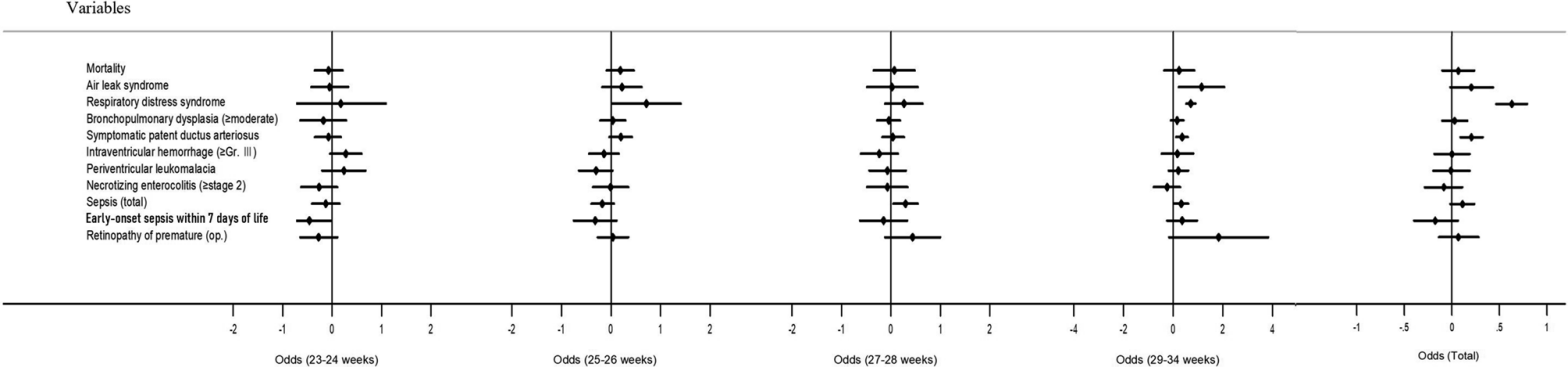
Adjusted odds ratios of mortality and morbidities associated with cesarean section (95% confidence interval).

Figure 2 illustrates the adjusted odds ratios (ORs) and 95% confidence interval (CI) for mortality and morbidity associated with CS in all the subjects and in the GA-stratified subgroup. Adjusted variables were GA, 5-min Apgar score, SGA, multiple pregnancies, inborn, and PIH.

The total adjusted ORs for mortality (OR 1.06, 95% CI 0.89–1.25) and morbidity due to BPD (OR 1.04, 95% CI 0.90–1.19), severe IVH (OR 0.99, 95% CI 0.82–1.18) and sepsis (OR 1.12, 95% CI 0.98–1.27), which showed significant reduction with CS than VD in univariate analyses, were not significantly reduced with CS in the multivariate analyses. The adjusted ORs for RDS (1.89, 95% CI 1.59–2.23) and symptomatic PDA (1.21, 95% CI 1.08–1.37) were significantly increased with CS than VD.

## Discussion

In this study, contrary to the significantly lower rates of total mortality and morbidity including sepsis and BPD with CS than VD, the GA-stratified mortality in the 25–26 GW subgroup and sepsis rates in the 27–28 GW and 29–34 GW subgroups were significantly higher with CS than VD. Further, in multivariate analyses, after adjusting for the other risk factors such as GA, the 5-min Apgar score, SGA, multiple pregnancies, inborn babies, and PIH associated with mortality and morbidity^14–16^, the adjusted ORs for mortality and morbidities including BPD, severe IVH and sepsis were not significantly reduced with CS than VD. However, the adjusted ORs for RDS and symptomatic PDA were significantly increased with CS than VD. Consistent with our data, Riskin et al.^8^ reported a population-based Israel Neonatal Network study demonstrating that CS was associated with a significantly lower mortality than VD. However, in the multivariate analysis, the delivery mode had no effect on mortality during the vertex presentation of VLBWIs. Overall, these findings suggest CS for VLBWIs was not associated with any survival or morbidity advantage with GA at 23–34 GW.

In this study, the CS rates were directly proportional to GA, with the lowest rate of 61% associated with 23–24 GW and the highest 84% observed in the 29–34 GW subgroup. These findings suggested that the obstetrician’s willingness to perform elective CS and the obstetric indications for CS were selection-biased according to the GA-dependent perceived outcomes of VLBWIs^11^. The greater than 15-fold mortality in the 23–24 GW than in the 29–34 GW subgroup, and the more than threefold higher severe IVH in the 23–24 GW than in 29–34 GW subgroup regardless of delivery mode suggest the enigmatic total but not GA-stratified mortality and morbidity advantages associated with CS in the present and other studies^6, 9, 17–19^. These findings might be primarily attributed to skewing of the dataset caused by higher absolute proportion of the 29–34 GW subgroup with the highest CS rate and the lowest risk of mortality and morbidity. Overall, these findings indicate that CS for VLBWIs cannot be routinely recommended in the absence of other obstetric or fetal indications^8, 20^.

The limitations of the present study relate to its uncontrolled observational design. However, in the absence of a recent RCT study, which might not be feasible, we believe that this prospective nationwide large cohort study might be the best alternative to investigate the association between delivery mode and outcomes in VLBWIs^8, 9^. Nonetheless, the study is strengthened by the inclusion of a large dataset enough to facilitate data stratification according to GA. Another study limitation relates to the lack of rationale for CS. High overall CS rate of 78%, which is substantially above the known obstetric and fetal indications for CS^4–8, 20^ suggests that CS was the most preferred delivery mode by obstetricians regardless of its indications for VLBWIs in this study. However, CS performed as an elective or emergency surgery may have a different effect on the neonatal outcome. Emergency CS performed mostly for critical fetal complications is associated with a higher frequency of adverse neonatal outcomes when compared with elective CS, which is most often attributed to maternal complications. Nevertheless, the study does not indicate whether the CS was performed as an elective or emergency operation.

## Methods

### Patients

The KNN database registry prospectively registered the clinical information of VLBWIs admitted to 67 voluntarily participating NICUs covering more than 80% of VLBWIs in Korea^12, 13^. Based on the enrolment criteria of KNN, only VLBWIs actively resuscitated in the delivery room and admitted to the NICU were registered in this study. Resuscitating infants at more than 24 GW is mandatory by law in Korea, but most Korean tertiary NICUs are currently willing to resuscitate infants up to 23 GW. Trained staff used a standardized operating procedure to collect demographic and clinical information. Informed consent forms were signed by the parents of the infants during enrolment in the KNN. The present study was performed in accordance with the ethical standards as laid down in the 1964 Declaration of Helsinki and its later amendments. The de-identified KNN data were approved by the Institutional Review Board (IRB) of Jeonbuk National University Hospital for further analysis and interpretation.

This nationwide cohort study database registry of the KNN consisted of 9,286 eligible VLBWIs at 23–34 GW between January 1, 2013 and December 31 2017. The VLBWIs were stratified according to GA into four subgroups: 23–24, 25–26, 27–28 and 29–34 GW (Fig. 1). We compared maternal and neonatal variables including GA, birth weight, gender, SGA, 1- and 5-min Apgar scores, maternal GDM and PIH in the total and GA-stratified 23–24, 25–26, 27–28, and 29–34 GW subgroups according to the delivery mode. We compared the mortality rates and major morbidities, including BPD, symptomatic PDA, severe IVH (grade ≥ 3), PVL, NEC, retinopathy of prematurity (ROP), and neonatal sepsis in the total population and the GA-stratified 23–24, 25–26, 27–28, and 29–34 GW subgroups according to the delivery mode.

### Definitions

We compiled a KNN database operation manual to define the patient characteristics. In the manual, GA was determined using the obstetric history based on the last menstrual period. SGA was defined as < 10 percentile birth weight of estimated GA. Chorioamnionitis was based on placental pathology^21^, and preterm premature rupture of membrane (PPROM) was defined as the rupture of membranes over 24 h before the onset of labor^22^. Moderate-to-severe BPD was defined as the use of more than supplemental oxygen at 36 GW, using the severity-based definition of the National Institutes of Health consensus^23^. Symptomatic PDA was defined by echocardiographic findings of patent ductus with predominant left-to-right shunt plus clinical symptoms of PDA including heart murmur, ventilator dependence, deteriorating respiratory status, increasing recurrent apnea, pulmonary hemorrhage and hypotension. IVH was defined as grade ≥ 3 according to the classification of Papile et al.^24^ PVL was defined as cystic PVL based on either head ultrasound or cranial magnetic resonance imaging scans performed at ≥ 2 weeks of age. NEC was defined as ≥ stage 2b according to the modified Bell criteria^25^. Early-onset sepsis was defined as a positive blood culture less than 7 days from birth in symptomatic infants suggestive of septicemia and more than 5 days of antibiotic treatment^26^. ROP was defined by treatment with anti-vascular endothelial growth factor, laser ablation, or surgery to prevent visual loss^27^.

### Statistical analysis

The characteristics of the study participants and their prenatal and neonatal morbidities are described as mean ± standard deviation for continuous variables, and as frequency and proportions for binary and categorical variables. Continuous variables were compared using Student’s t-test or Wilcoxon rank-sum test. Categorical variables were compared using the chi-square or Fisher’s exact test. Multiple logistic regression analysis was used to estimate the odds ratios (ORs) with 95% confidence interval (CI) adjusted for GA, 5-min Apgar scores, SGA, multiple pregnancies, inborn babies, and PIH. A *p*-value < 0.05 was considered statistically significant. Statistical analyses were performed using STATA version 14.0 (STATA Corp., College, TX, USA).

## Data Availability

Data availability was subject to the Act on Bioethics and Safety [Law No. 1518, article 18 (Provision of Personal Information)]. Contact for sharing the data or accessing the data is possible only through the data committee of Korean neonatal network (http://knn.or.kr) and after permission by the CDC of Korea. The contact information of the data access and ethics committees are Yun Sil Chang (cys.chang@samsung.com) and Jang Hoon Lee (neopedlee@gmail.com), respectively.
